# The influence of anticipated monetary incentives on visual working memory performance in healthy younger and older adults

**DOI:** 10.1038/s41598-020-65723-5

**Published:** 2020-06-01

**Authors:** Annamária Manga, Pál Vakli, Zoltán Vidnyánszky

**Affiliations:** 1 Brain Imaging Centre, Research Centre for Natural Sciences, Budapest, 1117 Hungary; 2 Department of Cognitive Science, Budapest University of Technology and Economics, Budapest, 1111 Hungary

**Keywords:** Cognitive ageing, Learning and memory, Reward, Human behaviour

## Abstract

Motivation exerts substantial control over cognitive functions, including working memory. Although it is well known that both motivational control and working memory processes undergo a progressive decline with ageing, whether and to what extent their interaction is altered in old age remain unexplored. Here we aimed at uncovering the effect of reward anticipation on visual working memory performance in a large cohort of younger and older adults using a delayed-estimation task. We applied a three-component probabilistic model to dissociate the reward effects on three possible sources of error corrupting working memory performance: variability in recall, misbinding of object features and random guessing. The results showed that monetary incentives have a significant beneficial effect on overall working memory recall precision only in the group of younger adults. However, our model-based analysis resulted in significant reward effects on all three working memory component processes, which did not differ between the age groups, suggesting that model-based analysis is more sensitive to small reward-induced modulations in the case of older participants. These findings revealed that monetary incentives have a global boosting effect on working memory performance, which is deteriorated to some extent but still present in healthy older adults.

## Introduction

Ageing has a negative impact on a variety of high-level cognitive functions, including working memory (WM)^1^. WM plays a crucial role in goal-directed behaviour, by performing the short-term maintenance and mental manipulation of information necessary for accomplishing our ongoing tasks^2,3^. The age-related impairment in WM may arise in the forms of capacity-reduction and decline in the filtering of relevant information^4^, diminution of the maintained representations’ resolution, and the increase in variability with which the memoranda are recollected, especially under high cognitive load^5,6^. Importantly, WM relies on the neural interactions within a network of distributed cortical and subcortical brain regions, including the dopamine neurotransmission mediated computations within the fronto-striatal circuitry (for a review see^7^). Therefore, it is reasonable to assume that age-dependent deterioration of the dopaminergic system^1,8,9^ might play an important role in the decline of WM performance in ageing.

In fact, dopamine neurotransmission is a key component of reward processing^10,11^. Reward-learning has an influence on the allocation of attention towards potentially rewarding stimuli via establishing associations between actions and corresponding reward outcomes, and among several other functions has been suggested to decline in ageing. Empirical evidence has shown that older adults are impaired compared to young participants on a wide range of tasks investigating reward processing, for example on monetary incentive delay task^12^, financial decision-making task^13^ or reward-based learning task with deterministic feedbacks^14^.

Furthermore, in line with the importance of motivation and effort allocation in cognitive performance, monetary incentives have been shown to affect higher-level cognitive functions, including WM. It has been repeatedly demonstrated that in young adults, reward improves WM performance (for reviews see^15,16^, and for recent empirical results see^17–21^). Taken together, the converging evidence supporting the beneficial effect of reward on WM function in young age thus raises the intriguing possibility that age-dependent decline in reward-based motivational processes might contribute to the deterioration of WM performance in old age.

In spite of the importance of this question, however, the number of empirical studies investigating the interaction of ageing, reward anticipation and cognition is limited, and their findings are highly inconsistent, in some cases even contradictory^16^. One line of empirical research has shown that the performance-enhancing effect of reward remains preserved in older adults, resulting in, for instance, comparable perceptual classification performance between younger and older adults for gain and loss associated stimuli compared to stimuli associated with neutral reward outcome^22^. Moreover, boosting effect of incentives in both younger and older adults has been observed on delayed recognition of pictures (i.e. episodic memory^23^), and on response latencies during a visual search task^24^.

In contrast, other studies have demonstrated a diminishing effect of reward on cognition in older adults, indicating faster response times in high-reward condition compared to low-reward condition in young (but not in old) participants on an incentivised reaction time task^25^ and on an indoor-outdoor classification task^26^. In addition, reward had no effect on episodic memory accuracy in older adults in a delayed recognition task using words as study material^27^. Furthermore, to our knowledge, the only study investigating the modulatory effects of reward anticipation on age-related changes in WM performance has also provided mixed behavioural results: WM accuracy was improved by reward in both younger and older adults, whereas reaction time was shortened by reward only in the case of young participants, measured on an n-back task with age-adjusted memory load^28^.

**Figure 1:**
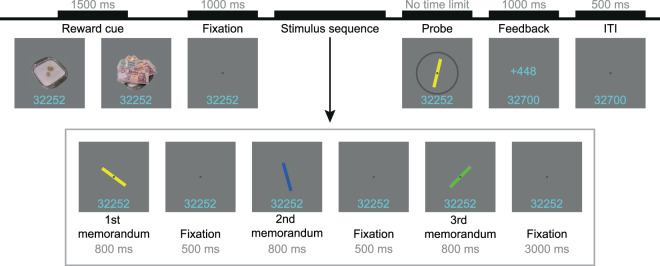
Schematic illustration of one trial of the incentivised visual WM task. Participants were instructed to memorise three sequentially presented coloured bars, and adjust the probe bar’s orientation using the keyboard to replicate the orientation of the target bar with the corresponding colour. At the beginning of each trial, either a small (on the left) or a large (on the right) reward cue indicated the reward value of the current trial. ITI stands for intertrial interval.

The current study aimed at uncovering the effect of monetary incentives on visual WM in a large group of younger and older adults using a WM paradigm that enables the dissociation of the different component processes underlying WM performance (see Fig. 1). We used a modified version of the continuous report task of Gorgoraptis and colleagues^29^, which allows a more precise measurement of the quality of WM representations compared to other commonly used paradigms testing WM via yes/no decisions (i.e. change detection tasks). The three-component probabilistic model^30^ applied on data acquired with delayed-estimation techniques assumes that inaccuracies in recall derive from three possible sources: variability in recall, misbinding of object features and random guessing (see^29–31^). Accordingly, analysis of these model components may provide the opportunity for a better understanding of the mechanisms underlying age-related changes in the reward-modulation of WM performance. Considering the notion that the effectiveness of cognitive functions shows great interindividual variability in old age^32,33^, a virtue of the current study is the large sample size, involving seventy-five healthy younger and seventy-five healthy older adults. Subjects performed an incentivised delayed-estimation task, where participants were instructed to memorise sequentially presented coloured bars with different orientations, and reproduce the orientation of the cued memoranda as accurately as possible using the keyboard. At the beginning of each trial, a reward cue indicated whether small or large monetary reward could be earned. The results revealed that monetary incentives improved the quality of visual WM representations in younger but not in older adults measured on a general indicator of WM performance (i.e. recall precision). However, investigation of the processes underlying recall performance derived from the three-component probabilistic model showed that the beneficial effect of reward was present on the model parameters in both younger and older adults, suggesting that monetary incentives have a global boosting effect on working memory performance, which is diminished to some extent but still present in healthy older adults.

## Results

### The effect of reward on visual WM performance in younger and older adults

#### Recall precision

**Figure 2:**
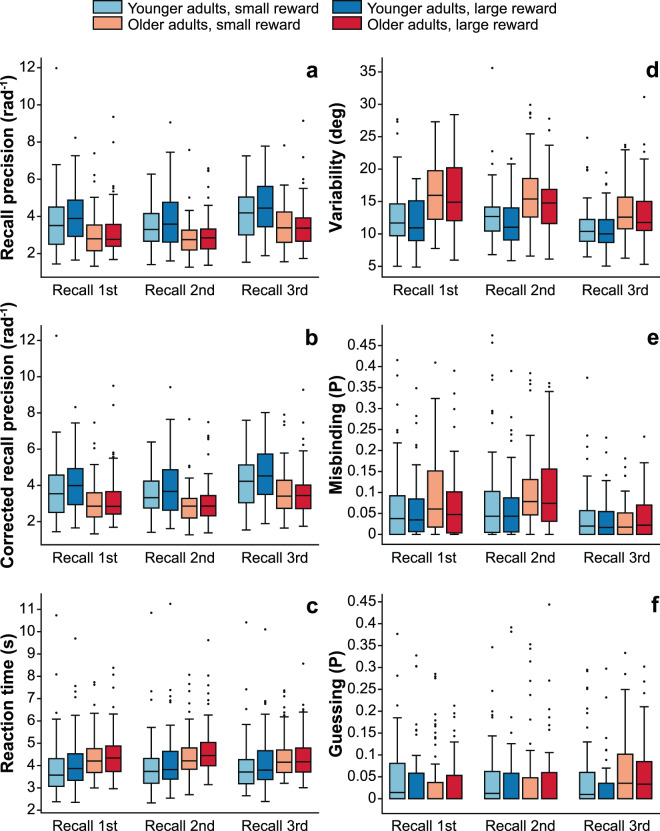
Box plots illustrate different indicators of visual WM performance separately for the two age groups and the two reward conditions (light blue box: younger adults, small reward; dark blue box: younger adults, large reward; light red box: older adults, small reward; dark red box: older adults, large reward) and the position of the target bar in the stimulus sequence (labelled below the x-axes). Panel a depicts recall precision, which is an overall measure of recall accuracy with higher values indicating better performance, while Panel b shows the recall precision corrected for sensorimotor precision measured on a separate task. On Panel c, the response times are depicted (it is important to note that response time was not limited), while Panels d, e and f illustrate the model parameters with lower values indicating better performance: Panel d shows the Gaussian variability in the recall of an item’s orientation (measured in degrees), Panel e shows the probability of reporting the orientation of a non-target item by mistake (feature misbinding), and Panel f shows the probability of reporting a random orientation (guessing). On each box, the central line indicates the median, the box limits indicate the lower and upper quartiles, and the whiskers indicate the extreme values not considered outliers. Outliers are marked with dots.

Our experiment was aimed at uncovering how reward affects visual WM performance in younger and older adults. The precision, with which participants recalled an item’s orientation varied as a function of the trial’s reward category. Reward had a significant effect on recall precision (see Fig. 2a and Table 1; mixed ANOVA, age group × reward × position, main effect of reward: F_1,143_ = 32.857, p = 5.664×10^−8^, η^2^_p_ = 0.187), resulting in higher recall precision on high-reward trials. Recall precision also varied as a function of age group (main effect of age group: F_1,143_ = 16.038, p = 9.939×10^−5^, η^2^_p_ = 0.101), with more precise answers in the young group. We observed a significant recency effect (main effect of position: F_1.71,246.01_ = 58.301, p = 1.293×10^−19^, η^2^_p_ = 0.288), with the highest precision when the third bar, and the lowest when the second bar was tested. Our results showed a significant interaction between age group and reward conditions (age group × reward interaction: F_1,143_ = 10.526, p = 0.002, η^2^_p_ = 0.069), indicating that age has an impact on how reward changes recall performance. Post-hoc comparisons of the two reward conditions separately in the two age groups revealed a significant reward effect in the case of younger adults (p = 1.939×10^−9^), and only a non-significant trend of the reward effect in older adults (p = 0.084), with more precise answers in the high-reward condition in both age groups. Further significant interactions between age group, reward and position factors were not found (age group × position interaction: F_1.71,246.01_ = 0.758, p = 0.454, η^2^_p_ = 0.005; reward × position interaction: F_1.71,246.01_ = 0.100, p = 0.903, η^2^_p_ = 6.944×10^−4^; age group × reward × position interaction: F_1.71,246.01_ = 1.805, p = 0.167, η^2^_p_ = 0.012).

**Table 1:** The table contains the mean values of the different measures of performance (recall precision, recall precision corrected for sensorimotor performance, reaction time, variability of recall, probability of misbinding and probability of guessing, and their standard deviations in round brackets.

		Recall precision (rad^−1^)	Corrected recall precision (rad^−1^)	Reaction time (s)	Variability of recall (deg)	Probability of misbinding	Probability of guessing
Main effect of REWARD	*Small reward*	3.447 (1.337)	3.517 (1.397)	4.169 (1.148)	13.698 (4.677)	0.069 (0.087)	0.048 (0.075)
	*Large reward*	3.710 (1.465)	3.793 (1.530)	4.336 (1.187)	12.914 (4.404)	0.061 (0.075)	0.039 (0.066)
Main effect of AGE GROUP	*Younger adults*	3.960 (1.491)	4.019 (1.541)	4.046 (1.243)	11.800 (3.709)	0.058 (0.080)	0.041 (0.069)
	*Older adults*	3.181 (1.194)	3.275 (1.290)	4.468 (1.047)	14.876 (4.825)	0.073 (0.082)	0.046 (0.072)
Main effect of POSITION	*Recall 1st*	3.496 (1.462)	3.560 (1.507)	4.226 (1.174)	13.975 (4.929)	0.072 (0.085)	0.041 (0.071)
	*Recall 2nd*	3.289 (1.291)	3.351 (1.346)	4.322 (1.217)	13.943 (4.618)	0.086 (0.094)	0.041 (0.073)
	*Recall 3rd*	3.950 (1.388)	4.054 (1.470)	4.210 (1.118)	11.999 (3.776)	0.038 (0.052)	0.048 (0.069)
AGE GROUP × REWARD interaction	*Younger adults, small reward*	3.756 (1.450)	3.807(1.496)	3.941 (1.233)	12.258 (4.039)	0.064 (0.091)	0.048 (0.073)
	*Younger adults, large reward*	4.163 (1.507)	4.230 (1.559)	4.151 (1.248)	11.342 (3.294)	0.052 (0.067)	0.034 (0.065)
	*Older adults, small reward*	3.125 (1.124)	3.214 (1.219)	4.407 (1.001)	15.198 (4.833)	0.075 (0.083)	0.047 (0.077)
	*Older adults, large reward*	3.237 (1.260)	3.337 (1.359)	4.530 (1.090)	14.553 (4.808)	0.071 (0.081)	0.044 (0.068)
AGE GROUP × POSITION interaction	*Younger adults, recall 1st*	3.845 (1.539)	3.900 (1.585)	4.015 (1.245)	12.184 (4.058)	0.063 (0.081)	0.046 (0.076)
	*Younger adults, recall 2nd*	3.662 (1.399)	3.709 (1.440)	4.089 (1.278)	12.368 (3.820)	0.072 (0.094)	0.041 (0.069)
	*Younger adults, recall 3rd*	4.372 (1.449)	4.448 (1.506)	4.034 (1.213)	10.847 (3.003)	0.039 (0.058)	0.036 (0.063)
	*Older adults, recall 1st*	3.133 (1.285)	3.206 (1.337)	4.446 (1.054)	15.842 (5.074)	0.080 (0.087)	0.036 (0.064)
	*Older adults, recall 2nd*	2.901 (1.038)	2.978 (1.129)	4.566 (1.103)	15.858 (4.815)	0.101 (0.092)	0.041 (0.078)
	*Older adults, recall 3rd*	3.510 (1.174)	3.643 (1.314)	4.393 (0.980)	13.200 (4.120)	0.037 (0.046)	0.060 (0.073)
REWARD × POSITION interaction	*Small reward, recall 1st*	3.377 (1.460)	3.435 (1.503)	4.141 (1.166)	14.344 (4.974)	0.080 (0.091)	0.046 (0.077)
	*Small reward, recall 2nd*	3.147 (1.155)	3.199 (1.189)	4.205 (1.168)	14.563 (4.843)	0.089 (0.101)	0.043 (0.074)
	*Small reward, recall 3rd*	3.817 (1.299)	3.917 (1.394)	4.162 (1.116)	12.186 (3.778)	0.039 (0.055)	0.054 (0.074)
	*Large reward, recall 1st*	3.616 (1.459)	3.685 (1.506)	4.312 (1.179)	13.606 (4.872)	0.064 (0.076)	0.036 (0.063)
	*Large reward, recall 2nd*	3.431 (1.403)	3.503 (1.474)	4.440 (1.258)	13.324 (4.310)	0.083 (0.086)	0.039 (0.072)
	*Large reward, recall 3rd*	4.082 (1.464)	4.191 (1.534)	4.257 (1.121)	11.813 (3.778)	0.037 (0.050)	0.041 (0.064)
AGE GROUP × REWARD × POSITION interaction	*Younger adults, small reward, recall 1st*	3.687 (1.625)	3.739 (1.676)	3.904 (1.266)	12.706 (4.535)	0.069 (0.091)	0.054 (0.081)
	*Younger adults, small reward, recall 2nd*	3.466 (1.237)	3.506 (1.270)	3.957 (1.241)	12.973 (4.072)	0.079 (0.109)	0.044 (0.066)
	*Younger adults, small reward, recall 3rd*	4.115 (1.406)	4.177 (1.453)	3.961 (1.206)	11.094 (3.184)	0.044 (0.065)	0.046 (0.072)
	*Younger adults, large reward, recall 1st*	4.003 (1.442)	4.061 (1.482)	4.125 (1.223)	11.662 (3.471)	0.058 (0.070)	0.037 (0.071)
	*Younger adults, large reward, recall 2nd*	3.857 (1.528)	3.911 (1.575)	4.220 (1.309)	11.764 (3.472)	0.066 (0.076)	0.037 (0.071)
	*Younger adults, large reward, recall 3rd*	4.629 (1.456)	4.720 (1.519)	4.106 (1.223)	10.600 (2.810)	0.034 (0.049)	0.026 (0.051)
	*Older adults, small reward, recall 1st*	3.053 (1.194)	3.119 (1.231)	4.387 (1.003)	16.052 (4.863)	0.091 (0.091)	0.038 (0.073)
	*Older adults, small reward, recall 2nd*	2.814 (0.962)	2.878 (1.010)	4.463 (1.034)	16.219 (5.050)	0.100 (0.091)	0.042 (0.082)
	*Older adults, small reward, recall 3rd*	3.507 (1.105)	3.646 (1.284)	4.372 (0.978)	13.324 (4.027)	0.034 (0.040)	0.062 (0.075)
	*Older adults, large reward, recall 1st*	3.213 (1.373)	3.292 (1.439)	4.506 (1.106)	15.633 (5.302)	0.070 (0.082)	0.034 (0.055)
	*Older adults, large reward, recall 2nd*	2.988 (1.108)	3.077 (1.235)	4.669 (1.167)	14.951 (4.516)	0.101 (0.093)	0.041 (0.073)
	*Older adults, large reward, recall 3rd*	3.512 (1.247)	3.640 (1.353)	4.414 (0.988)	13.077 (4.237)	0.041 (0.051)	0.057 (0.072)

The table contains mean values followed by standard deviation values in round brackets.

#### Individual differences in sensorimotor precision do not account for our findings

In addition to the memory performance per se, our experimental task required a fine sensorimotor co-ordination. To investigate whether individual differences in sensorimotor precision had an impact on recall performance, we analysed recall precision data corrected for individual sensorimotor errors (measured on a separate sensorimotor adjustment task). The analysis of WM performance corrected for sensorimotor precision yielded a similar pattern of results as observed in the case of uncorrected recall precision (see Fig. 2b and Table 1). The main effects of all three factors on the corrected recall precision values were significant (mixed ANOVA, age group × reward × position, main effect of reward: F_1,143_ = 32.241, p = 7.334×10^−8^, η^2^_p_ = 0.184; main effect of age group: F_1,143_ = 13.425, p = 3.490×10^−4^, η^2^_p_ = 0.086; main effect of position: F_1.68,242.09_ = 57.155, p = 6.934×10^−19^, η^2^_p_ = 0.284), with more precise answers in the large-reward condition, higher precision in the young group, and the highest precision for the third bar and the lowest precision for the second bar as a target. Age affected significantly the effect of reward condition on recall precision (age group × reward interaction: F_1,143_ = 9.799, p = 0.002, η^2^_p_ = 0.064), with significant reward effect in the group of young adults (p = 3.566×10^−9^) and a non-significant trend of the reward effect in the case of older adults (p = 0.077) revealed by the post-hoc comparisons. Further significant interactions between the three factors were not found (age group × position interaction: F_1.68,242.09_ = 0.351, p = 0.671, η^2^_p_ = 0.002; reward × position interaction: F_1.68,242.09_ = 0.128, p = 0.876, η^2^_p_ = 8.879×10^−4^; age group × reward × position interaction: F_1.68,242.09_ = 2.028, p = 0.135, η^2^_p_ = 0.014).

#### Reaction time

Participants responded significantly slower on high-reward trials than on low-reward trials (see Fig. 2c and Table 1; mixed ANOVA, age group × reward × position, main effect of reward: F_1,143_ = 46.010, p = 2.880 × 10^−10^, η^2^_p_ = 0.243). Age also affected significantly the response durations with slower responses in the case of older adults (main effect of age: F_1,143_ = 5.101, p = 0.025, η^2^_p_ = 0.034), just as position did, with the fastest responses when the third bar, and the slowest responses when the second bar was tested (main effect of position: F_1.63,234.28_ = 14.688, p = 9.101 × 10^−7^, η^2^_p_ = 0.093). The effect of age group on the reward-related deceleration of answers was present only as a non-significant trend (age group × reward interaction: F_1,143_ = 3.154, p = 0.078, η^2^_p_ = 0.022), while the interactions between age group and position, and also between reward and position turned out to be significant (age group × position interaction: F_1.63,234.28_ = 3.455, p = 0.033, η^2^_p_ = 0.023; reward × position interaction: F_1.63,234.28_ = 8.642, p = 2.386 × 10^−4^, η^2^_p_ = 0.057). There was no significant three-way interaction observed (age group × reward × position interaction: F_1.63,234.28_ = 0.303, p = 0.737, η^2^_p_ = 0.002).

#### Model components

In order to investigate the possible mechanisms underlying the beneficial effect of reward on recall precision, we applied a probabilistic model to the data. According to this model, errors in the visual WM recall precision stem from three possible sources: variability in the recall of the orientation of the target bar, reporting the orientation of a non-target bar (misbinding), and random guessing. Consistent with our results on recall precision, this analysis revealed a beneficial effect of reward on all three model components.

Our results showed a decrease in the recall variability of target stimuli in the case of high-reward trials compared to low-reward trials, indicating better visual WM performance in the high-reward condition (see Fig. 2d and Table 1; mixed ANOVA, age group × reward × position, main effect of reward: F_1,143_ = 22.764, p = 4.473 × 10^−6^, η^2^_p_ = 0.137). Age group also had a significant effect on the variability of recall (main effect of age group: F_1,143_ = 28.866, p = 3.073 × 10^−7^, η^2^_p_ = 0.168), with lower response variability in the case of young participants. Subjects’ answers showed a recency effect with the lowest recall variability when the third bar was tested and approximately similar performance for the first two bars (main effect of position: F_1.81,261.15_ = 47.340, p = 1.211 × 10^−17^, η^2^_p_ = 0.247). Age did not alter significantly the effect of reward on recall variability (age group × reward interaction: F_1,143_ = 0.688, p = 0.408, η^2^_p_ = 0.005). The interaction between age group and position of the target was significant (age group × position interaction: F_1.81,261.15_ = 4.020, p = 0.021, η^2^_p_ = 0.027), while the interaction between reward and position was not significant (reward × position interaction: F_1.81,261.15_ = 2.289, p = 0.105, η^2^_p_ = 0.016) just as the three-way interaction (age group × reward × position interaction: F_1.81,261.15_ = 0.352, p = 0.695, η^2^_p_ = 0.002).

Errors originating from misbinding of the object features showed a significant reward effect with lower probability of misbinding in the high-reward condition (see Fig. 2e and Table 1; mixed ANOVA, age group × reward × position, main effect of reward: F_1,143_ = 5.613, p = 0.019, η^2^_p_ = 0.038), and the lack of the influence of age on the probability of misbinding errors (main effect of age group: F_1,143_ = 2.110, p = 0.149, η^2^_p_ = 0.015). The recency effect occurred in the case of misbinding errors as well, with the least probability of misbinding when the third and the highest when the second bar was the target (main effect of position: F_1.56,224.40_ = 39.758, p = 1.542 × 10^−15^, η^2^_p_ = 0.216). Age did not alter significantly the effect of reward on the occurrence of misbinding errors (age group × reward interaction: F_1,143_ = 1.402, p = 0.238, η^2^_p_ = 0.010), although had an influence on the effect of position (age group × position interaction: F_1.56,224.40_ = 3.834, p = 0.024, η^2^_p_ = 0.026). No significant interactions were observed in the case of the target bar’s position and reward condition (reward × position interaction: F_1.56,224.40_ = 1.890, p = 0.154, η^2^_p_ = 0.013), or in the case of the two within-subject factors’ interaction with age group (age group × reward × position interaction: F_1.56,224.40_ = 1.842, p = 0.161, η^2^_p_ = 0.013).

There was a significantly lower probability of answers based on random guessing on high-reward trials (see Fig. 2f and Table 1; mixed ANOVA, age group × reward × position, main effect of reward: F_1,143_ = 4.345, p = 0.039, η^2^_p_ = 0.030). Age and position did not affect significantly the occurrence of errors resulting from guessing (main effect of age group: F_1,143_ = 0.433, p = 0.512, η^2^_p_ = 0.003; main effect of position: F_1.86,267.47_ = 1.219, p = 0.297, η^2^_p_ = 0.008). Age group did not influence the effect of reward on the probability of guessing (age group × reward interaction: F_1,143_ = 1.821, p = 0.179, η^2^_p_ = 0.013), the only significant interaction occurred between age group and position factors (age group × position interaction: F_1.86,267.47_ = 5.357, p = 0.005, η^2^_p_ = 0.036; reward × position interaction: F_1.86,267.47_ = 0.421, p = 0.648, η^2^_p_ = 0.003; age group × reward × position interaction: F_1.86,267.47_ = 0.158, p = 0.844, η^2^_p_ = 0.001).

In short, our results show that reward improves the quality of visual WM representations only in the group of younger adults when investigated on an overall measure of performance, however, analysis of the effect of reward on the model components showed that monetary reward decreases the variability of answers, the probability of misbinding of object features and the probability of guessing in younger and older adults as well.

### Role of individual differences in visual WM performance and reward effect

#### Intelligence, visual WM performance, and the reward effect

According to the assumption that age-dependent decline in reward-based motivational processes might contribute to the deterioration of WM performance in older adults, it is reasonable to investigate the relationship between the effect of reward on cognition and cognitive performance per se. Analysis of the relationship between the overall visual WM performance and intelligence revealed that recall precision correlated significantly and positively with Working Memory Index (WMI) of WAIS-IV (r_s71_ = 0.298, p_c_ < 0.05, 97.5% bootstrap confidence interval CI = [0.033, 0.517], number of bivariate outliers NO = 0) but not with Processing Speed Index (PSI) (r_s70_ = 0.209, p_c_ > 0.05, 97.5% CI = [-0.079, 0.460], NO = 1) in the young group. In the group of older adults, similarly to the young group significant positive correlations were observed in the case of recall precision and WMI (r_s67_ = 0.348, p_c_ < 0.05, 97.5% CI = [0.032, 0.607], NO = 0) but not in the case of recall precision and PSI (r_s65_ = 0.173, p_c_ > 0.05, 97.5% CI = [−0.136, 0.417], NO = 2).There was no significant correlation between the reward index of recall precision and intelligence neither in the younger group (WMI: r_s71_ = 0.086, p_c_ > 0.05, 97.5% CI = [−0.156, 0.327], NO = 0; PSI: r_s70_ = −0.048, p_c_ > 0.05, 97.5% CI = [−0.325, 0.215], NO = 1), nor in the older group (WMI: r_s67_ = 0.045, p_c_ > 0.05, 97.5% CI = [−0.224, 0.295], NO = 0; PSI: r_s66_ = 0.016, p_c_ > 0.05, 97.5% CI = [−0.263, 0.274], NO = 1). Furthermore, there was a lack of significant correlation between the reward index of recall precision and overall recall precision (recall precision data not split by conditions) as well, in both groups (younger adults: r_s68_ = 0.004, p_c_ > 0.05, 95% CI = [−0.237, 0.235], NO = 4; older adults: r_s64_ = −0.150, p_c_ > 0.05, 95% CI = [−0.413, 0.097], NO = 5).

#### Reward-elicited affective reactions and reward sensitivity

**Figure 3:**
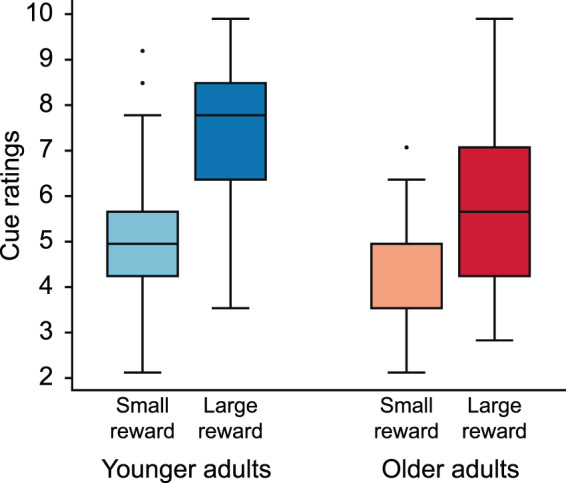
The box plot illustrates the subjective ratings of the cues indicating small and large reward in the incentivised WM task, separately in the two age groups (light blue box: younger adults, small-reward cue, mean (M) = 4.902, s.d. = 1.341; dark blue box: younger adults, large-reward cue, M = 7.463, s.d. = 1.405; light red box: older adults, small-reward cue, M = 4.183, s.d. 0.977; dark red box: older adults, large-reward cue, M = 5.677, s.d. = 1.844). Higher ratings indicate more positive self-reported attitude towards the cues. On each box, the central line indicates the median, the box limits indicate the lower and upper quartiles, and the whiskers indicate the extreme values not considered outliers. Outliers are marked with dots.

The comparison of self-reported affective reactions to reward cues indicating high and low reward between the two groups revealed that cue type had a significant main effect on the self-reported affective reactions (see Fig. 3; mixed ANOVA, age group × reward cue type, main effect of cue type: F_1,143_ = 239.129, p = 2.524 × 10^−32^, η^2^_p_ = 0.626) with higher rating of the cue indicating high reward. Age group also had a significant main effect on the affective reactions (main effect of age group: F_1,143_ = 40.439, p = 2.563 × 10^−9^, η^2^_p_ = 0.220) with higher rating of the cues in the group of younger adults. Furthermore, we observed significant interaction between the two factors (age group × reward cue type interaction: F_1,143_ = 16.558, p = 7.769 × 10^−5^, η^2^_p_ = 0.104). Post-hoc comparisons of the ratings of the two cues in the two groups revealed that participants in both groups rated more positively the high-reward cue than the low-reward cue (younger adults: p = 0; older adults: p = 4.439 × 10^−13^), with a greater difference between the two cues in the group of younger adults. We investigated whether the magnitude of the reward effect on recall precision is related to the affect elicited by the high-reward cues during the experiment, in the two age groups. In young participants, self-reported affective reactions showed a significant positive correlation with the reward index of recall precision (r_s70_ = 0.283, p_c_ < 0.05, 95% CI = [0.058, 0.487], NO = 2), while in older adults there was no significant correlation between the two variables (r_s69_ = −0.181, p_c_ > 0.05, 95% CI = [−0.384, 0.048], NO = 0). Investigation of the relationship between affective reactions to high-reward cue and the reward index of reaction time (obtained by subtracting the reaction time on low-reward trials from the reaction time on high-reward trials) revealed a positive correlation between the variables in the cases of younger adults (r_s70_ = 0.290, p_c_ < 0.05, 95% CI = [0.060, 0.496], NO = 2) and older adults (r_s68_ = 0.400, p_c_ < 0.05, 95% CI = [0.186, 0.569], NO = 1) as well. The analyses of BIS-BAS Scales revealed that - with the exception of the BAS Drive subscale – the answers of younger and older adults differed significantly from each other on the subscales of the questionnaire, with higher scores in the group of younger adults indicating higher sensitivity to punishment, and higher responsiveness to rewarding events and anticipated reward (see Table 2). However, none of the BIS-BAS subscales correlated significantly with the reward indices of recall precision or reaction time in the groups of younger and older adults (see Table 3).

**Table 2:** The table contains the results of the two-sample t-tests comparing the scores of younger and older adults on the subscales of BIS-BAS Scales, with group means and standard deviations in round brackets. BIS, BAS and df correspond to Behavioral Inhibition System, Behavioral Activation System, and degrees of freedom, respectively.

	df	t	*p*	Mean value (standard deviation)	
				Younger adults	Older adults
BIS	142	2.968	0.004	20.432 (3.836)	18.686 (3.174)
BAS Drive	142	1.008	0.315	11.473 (2.259)	11.086 (2.351)
BAS Fun Seeking	142	5.333	3.727 × 10^−7^	11.905 (1.761)	10.371 (1.687)
BAS Reward Responsiveness	142	6.405	2.068 × 10^−9^	17.662 (1.690)	15.557 (2.231)

**Table 3:** The table contains the results of skipped Spearman’s correlations between the subscales of BIS-BAS Scales, and the reward index of precision in younger adults (number of tested correlations (NC) = 4, corrected significance value (p_c_)> 0.05, 98.75% bootstrap confidence interval CI), reward index of reaction time in younger adults (NC = 4, p_c_ > 0.05, 98.75% CI) reward index of recall precision in older adults (NC = 4, p_c_ > 0.05, 98.75% CI), and reward index of reaction time in older adults (NC = 4, p_c_ > 0.05, 98.75% CI). None of the tested correlations were significant. BIS, BAS, NO and df correspond to Behavioral Inhibition System, Behavioral Activation System, number of bivariate outliers and degrees of freedom, respectively.

		BIS	BAS Drive	BAS Fun Seeking	BAS Reward Responsiveness
Younger adults, reward index of recall precision	NO	1	0	1	3
	df	71	72	71	69
	r_s_	−0.106	−0.077	0.121	0.038
	CI	[−0.366, 0.170]	[−0.368, 0.210]	[−0.206, 0.396]	[−0.273, 0.349]
Younger adults, reward index of reaction time	NO	0	0	5	0
	df	72	72	67	72
	r_s_	−0.163	0.003	0.026	−0.031
	CI	[−0.463, 0.115]	[−0.272, 0.313]	[−0.250, 0.337]	[−0.309, 0.265]
Older adults, reward index of recall precision	NO	3	0	0	1
	df	65	68	68	67
	r_s_	0.029	−0.033	−0.169	−0.121
	CI	[−0.282, 0.336]	[−0.361, 0.275]	[−0.440, 0.110]	[−0.419, 0.194]
Older adults, reward index of reaction time	NO	3	5	0	1
	df	65	63	68	67
	r_s_	0.011	0.129	−0.090	0.038
	CI	[−0.299, 0.327]	[−0.222, 0.439]	[−0.407, 0.225]	[−0.302, 0.370]

## Discussion

The aim of the current study was to examine the age-related changes in the modulatory effect of reward on visual WM performance on an incentivised delayed-estimation paradigm in a large sample of younger and older adults. In accordance with our expectations, reward increased the quality of visual WM representations measured on recall precision - which is an overall indicator of WM performance - only in the group of younger but not in the group of older adults. However, the group difference in the reward effect vanished when model components representing processes underlying WM recall were analysed. Monetary reward consistently reduced all three types of WM recall errors derived from our modelling, namely the variability of precision with which stimuli are recollected, the unintentional report of non-target stimuli, and random guessing, in both age groups. This is in accordance with the results on reaction time, showing that response time was increased by reward in both age groups. Additionally, our findings demonstrated a considerable recency effect in recall precision in both younger and older adults, with the highest accuracy when the last bar was tested in the three items long stimulus sequence. However, the effect of reward did not show a position-related change, indicating that there is no recency effect in the reward-modulation of WM. Taken together, our findings provide evidence that reward has a global boosting effect on WM performance in younger adults, and the beneficial effect of incentives is – despite a reduction - still present in older adults.

WM relies on the orchestrated activation of distributed cortical and subcortical brain regions, with a special emphasis on the dopamine-mediated fronto-striatal circuitry being pivotal in the gating of task-relevant stimuli into WM and in the retention of their memory representations^7^. Furthermore, dopamine is also involved in the motivational control of behaviour, i.e. in reward processing. Midbrain dopamine neurons have been shown to code reward prediction errors, the discrepancies between the expected reward value of an action based on our experiences and models of the environment, and the obtained reward value as a result of that action^11^. The ventral striatum, which supports the reward-related learning processes^15,34^, is richly innervated by midbrain dopaminergic neurons, and its activation - alongside ventromedial prefrontal cortex, another key region in reward monitoring - correlates with reward prediction errors^14^. An outstanding indicator of ventral striatum’s role in stimulus-reward learning is its activation while reward is anticipated, meaning the brain reward system is not only responding to reward reception, but to the cues pointing towards future rewards^34^. However, the dopaminergic system is strongly affected by ageing, incorporating the decline in the number of dopaminergic neurons in the substantia nigra^9^, the decrement in the density of the frontal and the reduction in the binding potential of the striatal dopamine receptors, and the decrease in the amount of the striatal dopamine transporters^8^. Based on the accumulated research on the role of the dopaminergic system in reward processing and cognition, it is reasonable to suggest that its age-dependent deterioration may affect both WM performance^4–6^ and reward processing^12–14^, moreover the two functions’ interactions as well. In spite of the importance of this question, the number of empirical studies investigating the interaction of ageing, reward anticipation and cognition is limited, and their findings are highly inconsistent, in some cases even contradictory^16^. While one line of empirical evidence shows that reward-modulation of cognition remains intact in older adults^22–24^, other line of research supports the idea of a diminishing reward effect in old age^25–27^. Our findings add new and robust evidence to the literature, that similarly to younger adults, reward has a boosting effect on WM performance in older adults as well. The absence of significant interaction between reward level and age group on model components and reaction time, and the presence of significant main effect of reward on these measures stand in favour of this claim. On the other hand, the significant interaction between age group and reward level on recall precision leads us to the assumption that the modulating effect of reward on performance undergoes a deterioration in old age. The reduction in reward processing is also supported by the results showing significant interaction between the two cue types and the two age groups in the perceived value of reward: the difference between the ratings of the two cues was significantly larger in the group of younger adults.

The difference in the effect of reward between markers of WM performance in older adults implies that these measures capture different functions underlying WM. Our finding that in younger adults monetary incentives improved the WM recall on each indicator of performance provides new evidence corroborating that reward-modulation of WM is a global process. However, the incoherence observed in older adults suggests that in old age, incentives do not affect each function equally, and the globality of the reward effect gets compromised in healthy ageing. The current findings are in line with a previous study^28^ investigating the age-related differences in the effects of incentives on WM, concluding that reward has a beneficial effect on WM in older adults, however not all aspects of performance are facilitated by incentives in ageing. Furthermore, our results have important implications related to the underlying processes of reduction in the motivational guidance of behaviour in ageing. The reduction in the perceived value of reward in older adults would suggest that decline in the processing of reward predicting cues per se is responsible for the diminished effect of reward on WM recall. However, our results on reaction time seem to argue against this possibility: participants in both age groups showed elevated reaction times in the high-reward compared to the low-reward condition, indicating an increased effort to perform well on trials offering high reward. On the other hand, our findings that the elevated response times on high-reward trials did not lead to increased recall performance in older adults raise the intriguing possibility that age-related changes in the motivational guidance of behaviour manifest in the reduced capability to convert effort to improvement of overall WM performance. It required a more sensitive modelling approach allowing the investigation of the different processes underlying WM recall to reveal that older adults still can profit from extra effort, similarly to that observed in the group of young participants.

An important question is whether the age-related deterioration of reward processing contributes to the decline observed on multiple domains of cognition in older adults, including WM. In accordance with the literature suggesting that age-related degradation is present on multiple fields of cognition (for a review on normal cognitive ageing see^35^), older subjects showed an overall reduction in performance regardless of reward condition on the visual WM task. However, we found no association between the magnitude of the reward effect and overall recall precision in visual WM or any of the intelligence measures of WAIS-IV. Thus, our results seem to argue against the contribution of age-related decline in the reward function to WM decline in old age. However, there are alternative explanations which might account for the absence of significant relationship between these functions. For example, the incentive manipulation in our task is only an indirect measure of the integrity of the brain reward system, therefore may reflect the interplay of several additional cognitive functions that might confound the correlation results.

To conclude, the present findings revealed that monetary incentives have a global, boosting effect on visual working memory performance in younger adults, which becomes diminished to some extent but is still present in healthy ageing.

## Methods

### Participants

75 healthy younger (44 female, mean age+/− standard deviation (s.d.) = 22.3 years +/− 2.7 years, age range: 18–33 years) and 75 healthy older (47 female, 67.5 years +/− 5.0 years, age range: 60–89 years), right-handed adults participated in the experiment. One younger adult and four older adults were excluded from the analysis due to insufficient performance (for further description of the exclusion criterion see Data analysis section), resulting in a sample of 74 younger (44 female, 22.3 years +/− 2.7 years, age range: 18–33 years) and 71 older (44 female, 67.5 years +/− 5.0 years, age range: 60–89 years) adults. Each volunteer had normal or corrected-to-normal vision (including colour-vision), with no reported history of neurological or psychiatric diseases. Subjects’ cognitive integrity was screened using Mini-Mental State Examination (MMSE)^36^, where each participant reached the criterion score of intact general cognition (younger adults: 29.45 mean MMSE, 0.75 s.d., 28–30 range; older adults: 28.82 mean MMSE, 0.82 s.d., 27–30 range). All subjects were remunerated for their participation and provided written, informed consent after the experiment was explained. Our research protocol was designed and conducted in accordance with the Hungarian regulations and laws, and was approved by the National Institute of Pharmacy and Nutrition (file number: OGYÉI/70184/2017). The experiment was carried out in the Brain Imaging Centre, Research Centre for Natural Sciences in Budapest, Hungary.

### Experimental tasks

#### Sensorimotor control task

The sensorimotor control task used was adopted from Burnett Heyes and colleagues^37^. In each trial, a randomly oriented target bar (2° × 0.3° of visual angle) of a randomly chosen colour (from the following pool: blue, green, yellow, red, magenta) appeared on a mid-grey background, and stayed on-screen until the end of the trial. Following a 500 ms delay, a randomly oriented probe bar (2° × 0.3° of visual angle, with identical colour to the target bar’s) surrounded by a dark-grey circle appeared above the target bar. Participants were asked to copy the orientation of the target bar as precisely as possible by rotating the probe bar using keys 7 and 8 on the numeric keypad of the computer. The orientations of the target and probe bars were chosen randomly and independently from a pool of angles ranging from −90 to 89 degrees of angle with a resolution of two degrees. Time provided to adjust the probe’s orientation was not limited, participants could proceed to the next trial by pressing the space key. Participants completed 25 trials of the sensorimotor control task, with 500 ms long inter-trial intervals.

#### Incentivised visual WM task

Participants performed a modified, incentivised version of the visual WM task employed by Gorgoraptis and colleagues^29^. During the entire task, each stimulus was presented centrally on a mid-grey background, with an overlaying dark-grey fixation spot at the centre of the screen with a diameter of 0.18° of visual angle. The fixation spot was visible throughout the trial. At the beginning of each trial, either a small-reward cue (a cash tray with three low value coins on it), or a large-reward cue (a cash tray covered by high value banknotes) was presented for 1500 ms indicating the reward category of the current trial. The cue was followed by a blank interval of 1000 ms during which only the fixation disc was present. Then, three stimulus bars (2° × 0.3° of visual angle) with different colour and orientation were presented consecutively, each displayed for 800 ms with 500 ms blank interstimulus intervals. The colours of the bars were randomly chosen in each trial from a pool of five colours (blue, green, yellow, red, magenta), with the criterion that the three bars presented within a trial should appear in different colours. The orientations of the bars were randomly chosen as well from a pool of angles ranging from −90 to 89 degrees with two degrees resolution, with a criterion that the orientation of the three bars within a trial should differ with at least 10 degrees of angle from each other. Following a delay period of 3000 ms after the offset of the third stimulus bar, a probe bar appeared on the screen surrounded by a dark-grey circle. The orientation of the probe bar was random, and its colour was identical to one of the previously presented three stimulus bars of the current trial. Subjects were instructed to memorise the colour and orientation of the three stimulus bars presented within the trial, and then recall the orientation of the stimulus bar whose colour was identical to the probe bar’s colour by rotating the probe bar using keys 7 and 8 on the numeric keypad of the computer. Volunteers were instructed to recall the orientation of the target stimulus bar as precisely as possible. Time provided to adjust the probe’s orientation was not limited, participants could finalise their answer by pressing the space key. Following space key press, a feedback (see next section) was present for 1000 ms. The intertrial interval, from the offset of the feedback to the onset of the next trial’s reward cue was 500 ms long.

At the beginning of the experimental session, participants were informed that they could earn points during the visual WM task, and at the end of the experiment the points collected would be converted to cash that they earn as a bonus in addition to a basic amount of 30€ (which was a compensation for their time). Subjects collected points in each trial of the visual WM task. The amount of points earned in each trial varied as a function of recall performance and the trial’s reward level which was indicated at the beginning of each trial by a reward cue. Half of the trials were low-reward trials, where the reciprocal of the recall error (angular difference between the original orientation of the target stimulus bar and the orientation of the manually adjusted probe bar) was multiplied by 5 points. The other half of the trials were high-reward trials, where the recall error’s reciprocal was multiplied by 500 points. As a result of this method, participants earned more points with more precise answers, with a maximum potential gain of 286 points in the case of low, and with a maximum potential gain of 28,648 points in the case of high-reward trials. Volunteers were informed about the points they won on the current trial via visual feedback presented in the centre of the screen (28 pixels text size, cyan colour) after the offset of the probe bar. In addition, a reward tracker (28 px, cyan) was always present at the bottom of the screen (with 7° of visual angle distance down from the centre) showing their cumulative score. Participants were not informed about the exchange rate between points and cash, but were ensured that the more points they collect, the more bonus they earn. At the end of the experiment, each participant earned equally a bonus of 6€ in order to avoid discrimination.

Trials were categorised, on the one hand, by the reward level of the trial (small vs. large reward), and on the other hand, by the position of the target stimulus bar in the stimulus sequence (1st vs. 2nd vs. 3rd bar was tested), resulting in six experimental conditions (small reward - recall 1st, small reward - recall 2nd, small reward - recall 3rd, large reward - recall 1st, large reward - recall 2nd, large reward - recall 3rd). Each participant completed 432 trials of the visual WM task in 12 runs, leading to 72 trials per condition. The sequence of trials from different conditions was randomised, and the trial number from different conditions was equalised across runs.

### Procedure

Our study consisted of two experimental sessions on separate days. In the first session (which lasted for approximately two hours), participants completed the Edinburgh Inventory^38^ to assess handedness, MMSE^36^ to assess general cognitive integrity, a colour naming task - where participants had to name the colours of the stimuli from the visual WM task to ensure they can discriminate the stimuli of the experiment -, and were evaluated for magnetic resonance imaging (MRI) eligibility. In order to assess the intellectual abilities of our participants, subtests of the Hungarian Wechsler Adult Intelligence Scale - Fourth Edition (Hungarian WAIS-IV)^39,40^ were completed. The following subtests were administered: Digit Span (forwards, backwards and inverse) and Arithmetic composing Working Memory Index (WMI); Symbol Search and Coding composing Processing Speed Index (PSI); Letter-Number Sequencing, Matrix Reasoning and Visual Puzzles. WAIS-IV data of one younger adult and two older adults were missing. In addition, volunteers completed Behavioral Pattern Separation Task—Object Version (BPS-O task)^41^ (data obtained in BPS-O task is not analysed in this paper, just as data obtained in Letter-Number Sequencing, Matrix Reasoning and Visual Puzzles subtests of WAIS-IV).

In the second - three hours long - session participants executed first the sensorimotor precision task, followed by the visual WM task. At the beginning of the visual WM task, participants saw a demo of the paradigm, and completed an additional training run (with no bonus collection). One run of the task lasted for approximately 10 minutes, participants had the opportunity to have short (maximum 1–2 minutes long) rests between the runs. In both sensorimotor and visual WM tasks stimuli were presented on a 24” Fujitsu LED monitor at a refresh rate of 60 Hz. Only one participant was present at a time, subjects sat in a dark room, and had to hold their head in a chin rest during the runs to ensure a constant viewing distance of 54 cm. Stimulus presentation and subject response registration was implemented in MATLAB R2015a (The Mathworks Inc., Natick, MA, USA) using PsychToolbox 3^42,43^. Following the visual WM task, participants had to rate, on seven-point Likert scales, the valence (from negative to positive) of their affective reaction and their level of arousal (from not aroused to highly aroused) elicited by the appearance of the reward cues indicating low-reward and high-reward probes during the experiment on a questionnaire adopted from Knutson and colleagues^44^. At the end of the session, volunteers completed the Hungarian adaptation of the Behavioral Inhibition System/Behavioral Activation System Scales (BIS-BAS Scales)^45,46^, with the following subscales: Behavioral Inhibition System (BIS), Behavioral Activation System (BAS) Reward Responsiveness, BAS Drive, and BAS Fun Seeking (BIS-BAS data of one older adult was missing). Subjects eligible for MRI completed two additional functional magnetic resonance imaging (fMRI) sessions, however the experimental procedures and results of the fMRI sessions are not discussed in this paper.

### Data analysis

In order to investigate the presence of any outliers in the two age groups, we calculated the mean of the recall error (the angular deviation between the orientation of the target bar and the response in each trial) in the case of each participant and applied the Carling’s modification of the boxplot rule using the Robust Correlation Toolbox^47^ for the labelling of outliers in mean error. One younger adult and four older adults were detected as outliers, with critically high mean of recall errors indicating poor task performance, and were thus excluded from all further analyses.

The reciprocal of the circular standard deviation of the recall error (calculated separately for each trial) was used as an overall measure of performance, called recall precision (with higher recall precision indicating better visual WM performance). In addition, to investigate possible mechanisms underlying performance, we applied a probabilistic model^30^ to the data. The Swap Model proposes that inaccuracies in the short-term retention and recall of visual object features can derive from three possible sources: a) Gaussian variability in the recall of an item’s orientation (measured in degrees); b) a certain probability of reporting the orientation of a non-target item by mistake (feature misbinding); and c) a certain probability of reporting a random orientation (guessing; with lower variability, misbinding and guessing indicating better performance)^29^. Maximum likelihood estimation was used to estimate the three parameters of the Swap Model for each subject and condition separately, using MemToolbox^31^ with ‘orientation’ extension in MATLAB (for a more detailed description of the model and the calculation of recall precision see^29–31^).

At the analyses of the effect of reward on performance we calculated recall precision and fitted the model separately for each subject and each condition. Reaction time was calculated for each subject and each condition as well, by subtracting the onset time of the response bar from the time of button press indicating the finalisation of the response in each trial. It is important to note, that since response time was not limited during the task, in the case of the current study longer reaction times do not mean weaker performance, but rather an intention of giving more precise, carefully wrought answers.

Since our experimental task required fine sensorimotor co-ordination, we investigated whether sensorimotor precision had a confounding effect on performance by correcting recall precision values with sensorimotor precision values obtained in a separate measurement. Similar to recall errors, sensorimotor adjustment errors were calculated as the angular differences between the target orientations and response orientations. Corrected recall precision was then calculated as the reciprocal of the square root of difference between the variances of recall errors and sensorimotor adjustment errors (for a more detailed description of the method and underlying theory see^37^).

The effect of age group, reward level and the target bar’s position in the stimulus sequence on the above measures (recall precision, recall variability, misbinding, guessing, reaction time and corrected recall precision) was investigated using mixed analyses of variance (ANOVAs) with age group (younger and older adults) as between-subject factor and with reward category (low and high reward) and serial position (1st, 2nd and 3rd) as within-subject factors with an alpha level of 0.05. In the case of significant age group × reward interaction, multiple comparison tests of the reward levels in the two age groups were performed applying the Bonferroni method. In cases of violations of sphericity (tested with Mauchly’s sphericity test), Greenhouse-Geisser correction was applied, and at the results section corrected degrees of freedom are reported.

In addition, we investigated whether overall visual WM performance showed a relationship with indices of intelligence, namely WMI and PSI (higher scores indicate higher cognition). To this end we calculated recall precision for each subject across all reward levels and serial positions. We assessed the relationship between recall precision, WMI and PSI separately in the two age groups using skipped Spearman’s correlations computed with the Robust Correlation Toolbox^47^. The calculation of the correlation values happened after the removal of the bivariate outliers (detected applying the Carling’s modification of the boxplot-rule) from the data. Bootstrap Confidence intervals (CI) were determined based on 1000 samples. Significance levels and CIs were adjusted using Bonferroni correction (number of tested correlations (NC) = 2, corrected significance value (p_c_) <0.05, 97.5% CI). Furthermore, we also tested the presence of a relationship between intelligence and the magnitude of the reward effect. In order to quantify the effect of reward on precision, we calculated recall precision separately for each subject and the two reward conditions (data was not split by the three target positions in the current analysis), and subtracted the precision on the low-reward condition from the precision on the high-reward condition to obtain a reward index. Then, we correlated this reward index of precision with WMI and PSI intelligence indices (skipped Spearman’s correlations, separately in the two age groups, NC = 2, (p_c_) <0.05, 97.5% CI). We also investigated whether the reward index of recall precision is related to the overall recall precision, via skipped Spearman’s correlations, separately in the groups of younger and older adults (NC = 1, (p_c_) <0.05, 95% CI).

To test whether there was a difference between the participants’ affective reactions to the cues indicating small and large reward – which were calculated as the sum of the ratings on valence/arousal scales of high reward and low reward, both sums divided by the square root of two (see^44^), with higher values indicating more positive valence and arousal – we compared the self-reported affective reactions to the small-reward and large-reward cues in the two age groups using mixed ANOVA with age group as between-subject factor and cue type (small-reward and large-reward cue) as within-subject factor (with an alpha level of 0.05, multiple comparison tests of the reward levels in the two age groups were performed applying the Bonferroni method). In order to investigate whether the magnitude of the reward effect of recall precision is related to the affective reaction elicited by the high-reward cue, we used skipped Spearman’s correlations separately in the younger and older adult groups (NC = 1, p_c_ < 0.05, 95% CI). We tested the relationship between the affective reactions to the high-reward cue and the reward index of reaction time – where the latter was calculated as the subtraction of reaction time on low-reward trials from the reaction time on high-reward trials – using skipped Spearman’s correlations separately in the two age groups (NC = 1, p_c_ < 0.05, 95% CI). In order to investigate whether there was a difference between younger and older adults in the scores reached on the subscales of BIS-BAS Scales, we used two-sample t-tests with an alpha level of 0.05. We also tested whether the subscales show relationship with the magnitude of the reward effect, using skipped Spearman’s correlations between the four subscales and the reward index of recall precision separately in the groups of younger and older adults (NC = 4, p_c_ < 0.05, 98.75% CI), and between the subscales and the reward index of reaction time separately in the younger and older adult groups (NC = 4, p_c_ < 0.05, 98.75% CI).

## Data Availability

The datasets generated and analysed during the current study are available from the corresponding author on reasonable request.
